# Peto’s Paradox: how has evolution solved the problem of cancer prevention?

**DOI:** 10.1186/s12915-017-0401-7

**Published:** 2017-07-13

**Authors:** Marc Tollis, Amy M. Boddy, Carlo C. Maley

**Affiliations:** Virginia G. Piper Center for Personalized Diagnostics, Biodesign Institute, Arizona State University, 727 E. Tyler St., Tempe, AZ 85287-5001 USA

## Abstract

The risk of developing cancer should theoretically increase with both the number of cells and the lifespan of an organism. However, gigantic animals do not get more cancer than humans, suggesting that super-human cancer suppression has evolved numerous times across the tree of life. This is the essence and promise of Peto’s Paradox. We discuss what is known about Peto’s Paradox and provide hints of what is yet to be discovered.

## What is Peto’s Paradox?

Peto’s Paradox is named after epidemiologist Richard Peto, who noted the relationship between time and cancer when he was studying how tumors form in mice. Peto observed that the probability of cancer progression was related to the duration of exposure to the carcinogen benzpyrene [1]. He later added body mass to the equation, when he wondered why humans both contain 1000 times more cells and live 30 times longer than mice, yet the two species do not suffer incredibly different probabilities of developing cancer [2]. Further, cancer was not a major cause of mortality for large and long-lived wild animals, despite the increased theoretical risks. How can this be?

## Why is it a paradox?

**Fig. 1.**
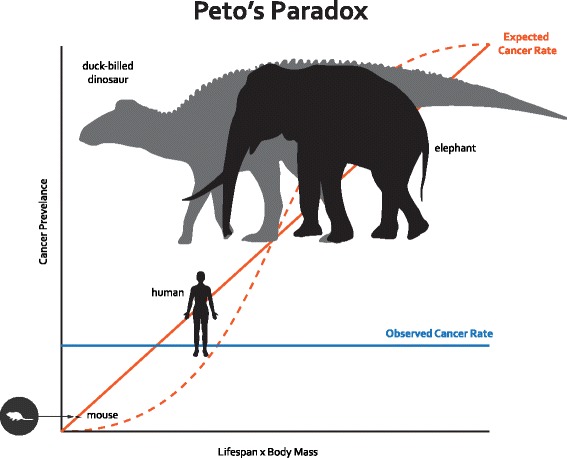
An illustration of Peto’s Paradox. Cancer is a disease of uncontrolled cell growth and division, and the risk of developing cancer increases with the number of cell divisions during the lifetime of an organism. Thus, the expected cancer rate for large and/or long-lived species is higher than for smaller short-lived ones. The *solid red line* indicates a linear relationship between cancer rate and (body mass)*(lifespan) and the *dashed red line* represents an approximation of the expected cancer rate assuming a model describing the probability of an individual developing colorectal cancer after a given number of cell divisions [4]. The *solid blue line* represents the observation that there is no relationship between cancer risk and (body mass)*(lifespan) [5]. For instance, cancer risk, which is 11–25% in the human population, is not vastly different between mice and humans. In contrast, cancer risk was estimated to be 5% in elephants [5]. Metastatic cancer was found in a duck-billed dinosaur [26], suggesting cancer was common enough in that lineage to be preserved in the fossil record, but not in other species of large dinosaurs. While adult body mass is approximately the same for the dinosaur and the elephant, duck-billed dinosaurs are thought to have had a shorter lifespan [28, 31]. This suggests that the trade-offs between reproduction and growth and cancer defense mechanisms [22] left these dinosaurs more susceptible to cancer than elephants

In a multicellular organism, cells must go through a cell cycle that includes growth and division. Every time a human cell divides, it must copy its six billion base pairs of DNA, and it inevitably makes some mistakes. These mistakes are called somatic mutations. Some somatic mutations may occur in genetic pathways that control cell proliferation, DNA repair, apoptosis, telomere erosion, and growth of new blood vessels, disrupting the normal checks on carcinogenesis [3]. If every cell division carries a certain chance that a cancer-causing somatic mutation could occur, then the risk of developing cancer should be a function of the number of cell divisions in an organism’s lifetime [4]. Therefore, large bodied and long-lived organisms should face a higher lifetime risk of cancer simply due to the fact that their bodies contain more cells and will undergo more cell divisions over the course of their lifespan (Fig. 1). However, a 2015 study that compared cancer incidence from zoo necropsy data for 36 mammals found that a higher risk of cancer does not correlate with increased body mass or lifespan [5]. In fact, the evidence suggested that larger long-lived mammals actually get *less* cancer. This has profound implications for our understanding of how nature has solved the cancer problem over the course of evolution.

## How does one go about solving the paradox?

From one perspective, the solution to Peto’s Paradox is quite simple: evolution [6]. When individuals in populations are exposed to the selective pressure of cancer risk, the population must evolve cancer suppression as an adaptation or else suffer fitness costs and possibly extinction. But that only tells us that evolution has found a solution to the paradox, not how those animals are suppressing cancer. Discovering the mechanisms underlying these solutions to Peto’s Paradox requires the tools of numerous subfields of biology including genomics, comparative methods, and experiments with cells. For instance, genomic analyses revealed that the African savannah elephant (*Loxodonta africana*) genome contains 20 copies, or 40 alleles, of the most famous tumor suppressor gene TP53 [5, 7]. The human genome contains only one TP53 copy, and two functional TP53 alleles are required for proper checks on cancer progression. When cells become stressed and incur DNA damage, they can either try to repair the DNA or they can undergo apopotosis, or self-destruction. The protein produced by the TP53 gene is necessary to turn on this apoptotic pathway. Humans with one defective TP53 allele have Li Fraumeni syndrome and a ~90% lifetime risk of many cancers, because they cannot properly shut down cells with DNA damage. Meanwhile, experiments revealed that elephant cells exposed to ionizing radiation behave in a manner consistent with what you would expect with all those TP53 copies—they are much more likely to switch on the apoptotic pathway and therefore destroy cells rather than accumulate carcinogenic mutations [5, 7].

## How many different solutions to Peto’s Paradox are there?

There are likely many solutions to Peto’s Paradox in nature, because large body size has evolved independently so many times across the history of life. We know that whales did not evolve the extra copies of TP53 like elephants [8, 9]. In fact, there is no evidence that whales evolved extra copies of any tumor suppressor gene—even the gigantic bowhead whale (*Balaena mysticetus*), which has a lifespan of over 200 years [9]. In populations, large body size is often associated with higher fitness, conferring greater access to resources or mates and better predator avoidance. It is not surprising, therefore, that large body size has evolved again and again throughout evolutionary time—a trend first realized in the fossil record and known as Cope’s Rule [10]. Cope’s Rule applies widely across life from diverse marine taxa [11] to the extinct giant dinosaurs [12]. Large body size has evolved independently in 10 of the 11 placental mammalian orders [13]: think polar bears and hippopotami, walruses and giraffes, elephants and whales. Since many lineages faced the trade-off between large body size and cancer risk during their evolution, there have likely been many different pathways in which cancer suppression has evolved.

## What solutions to the paradox do we know?

In the example of the elephant given above, greater tumor suppressor gene redundancy provides better checks on the cell cycle, in effect ‘blowing up’ cells with DNA damage and preventing cancer [5, 7]. Larger animals have a lower metabolic rate than smaller ones, and the mutagenic agents supplied by fast metabolisms—most notably reactive oxygen species that can damage DNA—are simply less common in large animals. This may also resolve Peto’s Paradox in some species. Some exceptionally long-lived rodents such as naked mole rats and blind mole rats are famous for having very low cancer rates. In the case of naked mole rats (*Heterocephalus glaber*), this seems to be due to a form of hyaluronic acid and a super-sensitive CDKN2A tumor suppressor pathway that suppresses proliferation of naked mole rat cells [14, 15]. Blind mole rats (*Spalax judaei* and *Spalax golani*) have a different mechanism of cancer suppression. Over-proliferation of blind mole rat cells triggers massive necrotic cell death that destroys both the proliferating cells and their neighbors [16].

There are a variety of hypotheses for other potential solutions to Peto’s Paradox. One attractive hypothesis is the prediction of ‘hypertumors’ [17]: bigger tumors in bigger animals take longer to grow and are susceptible to ‘cheater’ cells, which can take advantage of the tumor’s angiogenic properties and lower the fitness of the whole tumor. The effect of hypertumors could be to lower the overall lethality of cancer in the bodies of large animals. Other potential solutions could be: large animals have increased immunocompetence with better surveillance and attacking of neoplastic cells, or they may have shorter telomeres which would limit the number of cell divisions and thus the risk of cancer. However, these solutions have not yet been observed in large-bodied species and more research in these areas is needed.

## How can you translate a solution in some other species to prevent cancer in humans?

Ideally, comparative studies could highlight potential targets where the genetic mechanisms underlying cancer suppression in one species could be transferred to another, with clinical implications. For instance, it was found that genetically altering mice to overexpress a form of the TP53 protein conferred a cancer-suppressive phenotype; however, these mice also displayed a premature ageing phenotype [18]. Surprisingly, another study created ‘super p53’ mice which contained extra copies of the TP53 gene—similar to the elephant genome—under their normal promoters, and these mice revealed an enhanced DNA damage response and cancer suppression without the ageing effect [19]. Work is now underway to develop medicines based on the TP53 pathway. While the search for solutions to Peto’s Paradox across a diversity of species is still in progress [5, 7, 9, 20], it will no doubt require substantial effort to translate recent discoveries into effective therapies for humans.

## But wait, why haven’t all animals evolved extra tumor suppression mechanisms?

Cancer is a potential problem for all multicellular life and there is no expectation that a species should be completely cancer free; in fact, elephants still get cancer—about 5% of deaths in zoos according to one study [5]. Cancer has also been found in whales [21]. There are a few potential reasons that cancer is still a problem for multicellular animals. First, cancer defense mechanisms, such as DNA repair, cell cycle control, and immune function, can be costly. There are likely energetic trade-offs between cancer suppression and other important life history components, such as reproduction and growth [22]. Cancer is a disease of ageing populations both because there is weaker selection to avoid problems after reproduction [23], and because it takes time to accumulate all the mutations necessary to cause a cancer. For animals that are short-lived (such as mice), it doesn’t make much sense to invest much in cancer defense mechanisms. These animals are more likely to die of other extrinsic causes (such as predators) than of cancer. Second, benefits early in life that increase an organism’s fitness may lead to disease susceptibility later in life, an evolutionary term called antagonistic pleiotropy [24]. For example, there may be a genetic variant that allows an organism to get big fast—increasing its mating potential and decreasing the likelihood it will be killed by a predator—but this same genetic variant may also lead to cancer susceptibility as the animal ages.

## Did dinosaurs get cancer?

Birds evolved from a lineage of theropod dinosaurs during the Jurassic Period and they get cancer today [20], so we can say that, yes, absolutely, dinosaurs did and still do get cancer. Cancer incidence in extant birds is lower than in mammals [25], and the reasons for this are not well known and are worthy of future study. There is a fossil record of cancer for extinct non-avian dinosaurs as well; one study examined >10,000 vertebrae from >700 individuals across the dinosaur phylogeny [26]. The study concluded that the only examined non-avian dinosaurs with bone neoplasms were large, elephant-sized herbivores classified as hadrosaurs, also known as duck-billed dinosaurs, including one instance of metastatic cancer in a caudal vertebrae (tail bone) from an individual *Edmontosaurus*. A later study described a set of benign neoplasms in a titanosaur [27], which was a member of the sauropod family that included the largest terrestrial animals in Earth’s history. Whether the exclusivity of non-avian dinosaurian malignant cancers to hadrosaurs is due to a genetic component or some vagary or artifact of the fossil record is not known. However, similar sample sizes across many dinosaur clades were examined, so it appears to be statistically significant, and cancer may have occurred often enough in hadrosaurs so that some affected individuals became fossilized and eventually observed by paleontologists.

## Why did hadrosaurs get so much cancer but not other large dinosaurs?

The answer may lie in life history differences. Elephants have slow life histories, taking decades to reach adult size and investing heavily in the rearing of their offspring. It makes sense that natural selection led to the evolution of elephants with a means to suppress cancer, because only those that could suppress cancer lived long enough and grew large enough to out-compete their rivals. Can we apply this logic to extinct dinosaurs? The fact that one out of 16 *Edmontosaurus* specimens had metastatic cancer [26] suggests that cancer occurred at a rate sufficient to ensure preservation in the fossil record. Fortunately, some species of hadrosaur—including *Edmontosaurus*—left behind rich fossil deposits, giving paleontologists a window into hadrosaurian demographics [28, 29]. Hadrosaurs lived very different lives to our extant mammalian giants. They laid many eggs at a time in huge nests, suggesting greater reproductive output, and grew very rapidly, with some species reaching skeletal maturity in as little as 8 years. In addition, hadrosaurs may have had much shorter lifespans than modern gigantic mammals, with senescence beginning soon after skeletal maturity and some fossil deposits lacking any adults over ~16 years of age [28]. High reproductive output, rapid growth rates, and large body sizes may have been traits necessary for species survival during the Late Cretaceous Period ~70 million years ago, when predators like tyrannosaurs roamed the ancient floodplains. Perhaps the trade-offs associated with these traits, such as lack of sufficient DNA repair mechanisms [22], left hadrosaurs susceptible to cancer once reaching a certain size. Other large dinosaurs may have had slower life history strategies and so were under selection to evolve more effective cancer suppression mechanisms.

## Why should I care about Peto’s Paradox?

If cancer suppression has repeatedly evolved as a trait, then basic research into the life history, genomics, and cell biology of many different organisms can ultimately lead to better therapeutic strategies. If Peto’s Paradox holds across the tree of life, and large long lived organisms do in fact get less cancer than their body size warrants, then it implies numerous mechanisms exist to prevent neoplastic progression and battle cancer. Every time we discover a potential mechanism for cancer suppression in a species, there is the chance that we can find new therapeutic targets and approaches to cancer prevention to save human lives. Cancer has been part of the story of the evolution of multicellularity [29], and it is obvious that many lineages have evolved ways to cope with this disease. Now we are living in the midst of Earth’s sixth mass extinction, with extinction rates possibly 1000 times the historical rate [30]. Investigations into Peto’s Paradox can help cancer prevention research as well as foster an appreciation for biodiversity and the need to conserve species across the planet.
